# Revealing Individual Lifestyles through Mass Spectrometry Imaging of Chemical Compounds in Fingerprints

**DOI:** 10.1038/s41598-018-23544-7

**Published:** 2018-03-26

**Authors:** Paige Hinners, Kelly C. O’Neill, Young Jin Lee

**Affiliations:** Department of Chemistry, Iowa State University, Ames, IA 50011 USA

## Abstract

Fingerprints, specifically the ridge details within the print, have long been used in forensic investigations for individual identification. Beyond the ridge detail, fingerprints contain useful chemical information. The study of fingerprint chemical information has become of interest, especially with mass spectrometry imaging technologies. Mass spectrometry imaging visualizes the spatial relationship of each compound detected, allowing ridge detail and chemical information in a single analysis. In this work, a range of exogenous fingerprint compounds that may reveal a personal lifestyle were studied using matrix-assisted laser desorption/ionization mass spectrometry imaging (MALDI-MSI). Studied chemical compounds include various brands of bug sprays and sunscreens, as well as food oils, alcohols, and citrus fruits. Brand differentiation and source determination were possible based on the active ingredients or exclusive compounds left in fingerprints. Tandem mass spectrometry was performed for the key compounds, so that these compounds could be confidently identified in a single multiplex mass spectrometry imaging data acquisition.

## Introduction

Fingerprints consist of ridges and valleys that form a pattern unique to the individual. The sweat and chemical residues present on a finger can leave ridge detail, or a latent fingerprint, on objects and surfaces encountered by the fingers. Latent fingerprints have provided evidentiary value in forensic investigations for over a century, mainly as a means of identification through pattern comparison. As technology and science have advanced, so has the collection and interpretation of evidence within latent fingerprints. Chemical compounds in the residue contain detailed information about the individual depositing the fingerprint. Chromatography and mass spectrometry coupled methods, such as liquid chromatography-mass spectrometry (LC-MS) and gas chromatography-mass spectrometry (GC-MS), provide chemical fingerprint information, but typically require multiple fingerprints with no spatially relevant information^1–3^.

Surface mass spectrometry techniques with imaging capabilities supply chemical and spatial information in a single fingerprint analysis. Various ionization methodologies were used for mass spectrometry imaging (MSI) of fingerprints including secondary ion mass spectrometry (SIMS)^4–6^, desorption electrospray ionization (DESI)^7,8^, desorption ionization on porous silicon (DIOS)^9^, desorption electro-flow focusing ionization (DEFFI)^10^, and matrix-assisted laser desorption/ionization (MALDI)^11–15^. A challenge to compound identification in MSI is the lack of chromatographic separation and corresponding retention time information. We have previously developed a “multiplex MSI” technique that allows for simultaneous data acquisition of high-resolution mass spectrometry (HRMS) and tandem mass spectrometry (MS/MS) to enable chemical composition analysis and structural elucidation of each compound^16^. The multiplex MSI technique was successfully demonstrated in fingerprint analysis, allowing fingerprint chemicals to be confidently identified in a single analysis^17^.

A broad range of endogenous and exogenous fingerprint chemicals have been targeted using MALDI-MSI. Endogenous compounds, those naturally excreted from the human body, include compounds like amino acids, fatty acids, peptides, proteins, and triacylglycerols (TGs)^12,18–21^. Researchers have utilized endogenous compounds to potentially differentiate subsets of people or identify the age of the fingerprint^4,8,12,18,20^. Exogenous compounds are those present on the fingerprint from various forms of contamination, or any chemical present that is not naturally excreted from the human body. The most studied exogenous compounds in latent fingerprints are illicit drugs and explosives. They are topics of interest in criminal investigations, making them desirable sets of compounds to study^7,9–11,22^. While the importance of exogenous compounds has been recognized with drugs and explosives, the full potential of the vast range of other exogenous compounds has yet to be explored.

We are in constant contact with diverse chemicals in our daily life. Dorrestein and colleagues recently demonstrated that the lifestyle of an individual can be revealed based on chemical compounds obtained from the hands or cell phones^23,24^. Here, we hypothesize that the lifestyle of an individual can be characterized by many types of exogenous chemicals left in fingerprints. In this work, consumer products, foods, and alcohols were investigated as sources of exogenous compounds in latent fingerprints. The variations in the chemicals present were compared for brand and type determination. The presence of a single compound may not be sufficient to provide detailed information about an individual, but the compilation of multiple compounds present in a latent fingerprint may give insight into one’s lifestyle.

## Results and Discussion

### Matrix Selection

The first step of this study was to find optimum matrices for the compounds of interest. Two organic matrices ((α-cyano-4-hydroxycinnamic acid (CHCA) and 2,5-dihydroxybenzoic acid (DHB)) and two metal nanoparticles (gold and silver) were explored for optimum ionization across a broad range of compounds. CHCA is often used for endogenous and exogenous fingerprint analysis via MALDI-MSI^11,19^ and DHB has shown promise with TGs^12^. However, organic matrices typically have significant matrix contamination in the low mass range, suppressing many fingerprint chemicals. Most nanoparticles have minimal to no matrix background and are utilized to avoid matrix contamination peaks. Additionally, as they do not crystallize, they can be homogeneously applied to the fingerprints or sample tissues, in this case via uniform sputter coating.

CHCA and DHB were first utilized for the sunscreen compounds but did not efficiently ionize all six active ingredients. The gold and silver nanoparticles were sputter coated and did ionize all six sunscreen target compounds with varying ionization efficiencies. Silver offers the additional advantage of adduct formation, i.e., [M + Ag]^+^, useful in the analysis of other exogenous compounds, some of which were seen only as the silver adduct. Hesperidin, hesperetin, ethyl palmitate, ethyl myristate, and glycerol formal are examples of compounds only seen as the silver adduct. Silver adduct formation is particularly useful for hydrophobic compounds that do not otherwise ionize. In the analysis of food oils, DHB was used as the matrix, as it can best ionize TG compounds^12,25^.

### Brand Comparison in Bug Spray and Sunscreen

**Figure 1:**
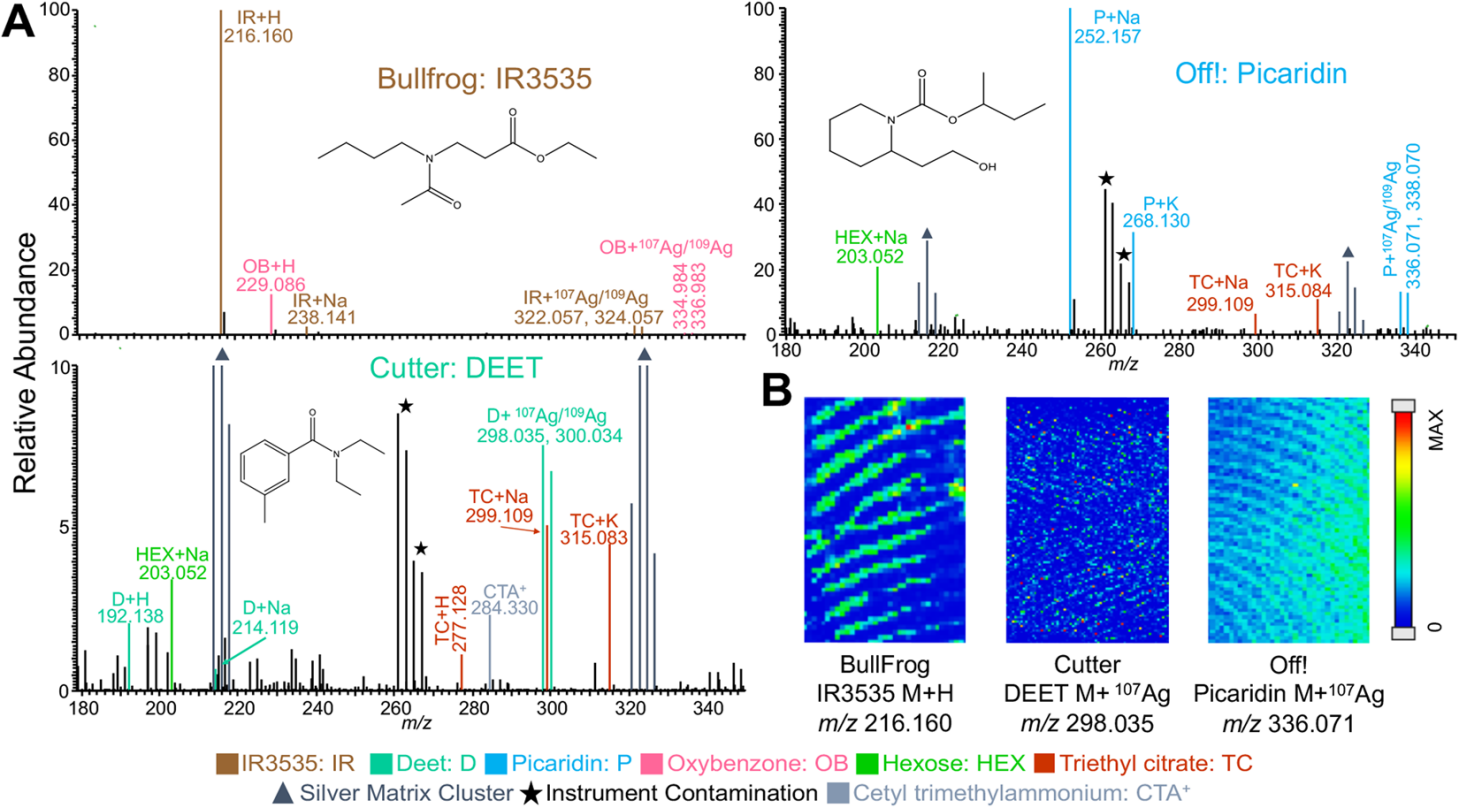
(**A**) Representative positive mode mass spectra of fingerprints containing three bug spray brands (BullFrog, Cutter, and OFF!) by MALDI-MSI with silver sputter. (**B**) Chemical images of the three active ingredients.

Bug spray and sunscreen are two consumer products indicative of an outdoor lifestyle. Active ingredients are listed on the label for both products, which allows brand comparison based on the differences in active ingredients among brands. BullFrog (active ingredient: insect repellant 3535 (IR3535)), Cutter (active ingredient: N,N-diethyl-m-toluamide (DEET)), and OFF! (active ingredient: Picaridin) were the bug sprays compared in this study. MALDI mass spectra of each brand obtained from deposited fingerprints are shown in Fig. 1A. The brand of origin could be easily correlated based on the color coded active ingredients. All three active compounds are tertiary amines and ionized as proton ([M + H]^+^), alkali ([M + Na]^+^, [M + K]^+^), and/or silver adducts ([M + ^107^Ag]^+^, [M + ^109^Ag]^+^) in positive mode. BullFrog is composed of a mixture of bug spray and sunscreen compounds. In BullFrog, oxybenzone, a UV filter in many sunscreens, is the next most abundant compound after IR3535. The intensity of each adduct for the three active ingredients was extracted and normalized to the most abundant compound for a graphical comparison of the bug sprays (Fig. S1). Fingerprint chemical images of each active ingredient were extracted from separate fingerprints to display the ridge detail in the contaminated prints (Fig. 1B). When active ingredients of bug spray are present in a latent print, they can be related to the brand of origin, then compared to items in the suspect’s possession, or may be used to narrow down the persons of interest.

**Figure 2:**
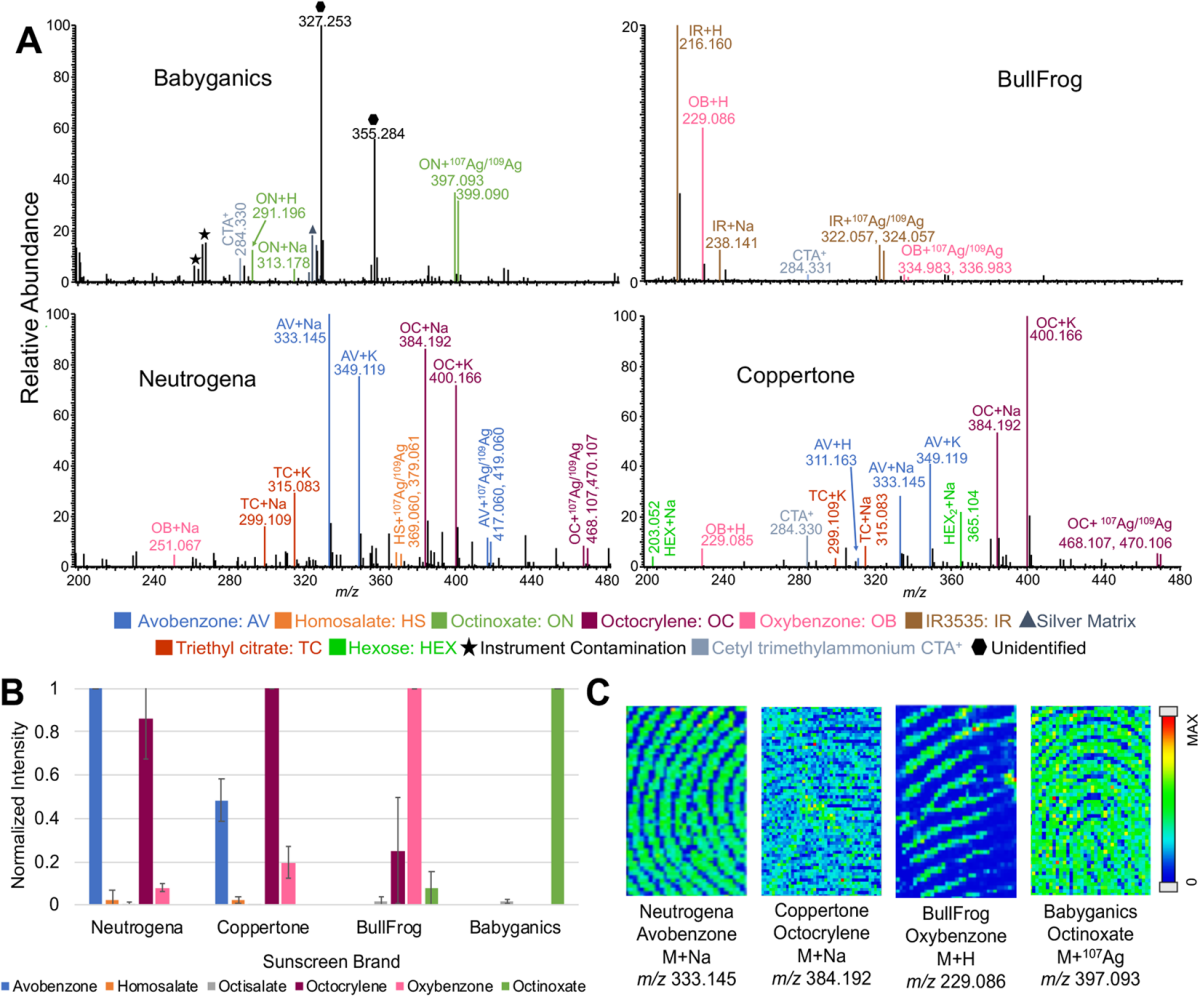
(**A**) Representative positive mode mass spectra of four sunscreen brands (Babyganics, BullFrog, Neutrogena, and Coppertone) by MALDI-MSI with silver sputter. (**B**) Comparison of each active ingredient across the four brands. The adduct intensities are summed for each active ingredient and normalized to the most abundant compound. Error bars show the standard deviation from three replicates. (**C**) Chemical images of the four sunscreen active ingredients used in brand determination.

Other miscellaneous ingredients that are found in Fig. 1A include a hexose, triethyl citrate, and cetyl trimethylammonium ion. None of which are active ingredients and are not useful in distinguishing the brands of bug spray. Cetyl trimethylammonium ion is most likely from a common surfactant used in many consumer products. Sugars, specifically hexose (e.g., glucose), are regularly found as exogenous compounds of many sources in fingerprints (see later section for citrus fruits and alcohols). In this work, triethyl citrate, an ester of citric acid, has also been identified in other consumer products and foods. Triethyl citrate is a common plasticizer used in consumer products. The same inactive ingredients are also found in sunscreen (Fig. 2A).

Each brand of sunscreen contains multiple active components as summarized in Table S1 and the mass spectra are shown in Fig. 2A with color-coded active ingredients. Two sunscreen brands, BullFrog and Babyganics, could be easily distinguished by the unique active ingredients: oxybenzone and IR3535 (an insect repellent) for BullFrog and octinoxate for Babyganics. Octinoxate is also present in BullFrog but in very low abundance. At a glance, Neutrogena and Coppertone are not easily distinguished as they both have avobenzone and octocrylene as the major active ingredients. However, they can be distinguished based on the relative intensities of each compound. In Neutrogena, the two active ingredients have similar intensities with avobenzone being slightly higher, but in Coppertone octocrylene has a much higher abundance. It was reproducible across replicates, as their normalized intensities are compared in Fig. 2B. The *m/z* 327 and *m/z* 355 peaks in Babyganics are likely related to short chain diacylglycerol (DG) species but could not be confidently identified. Avobenzone, octocrylene, oxybenzone, and octinoxate are the most useful sunscreen compounds for brand determination. Chemical images of each relevant sunscreen compound from individual fingerprints are shown in Fig. 2C. As with bug spray, the sunscreen brand can be compared to an individual’s possessions to assist in lifestyle compilation.

**Figure 3:**
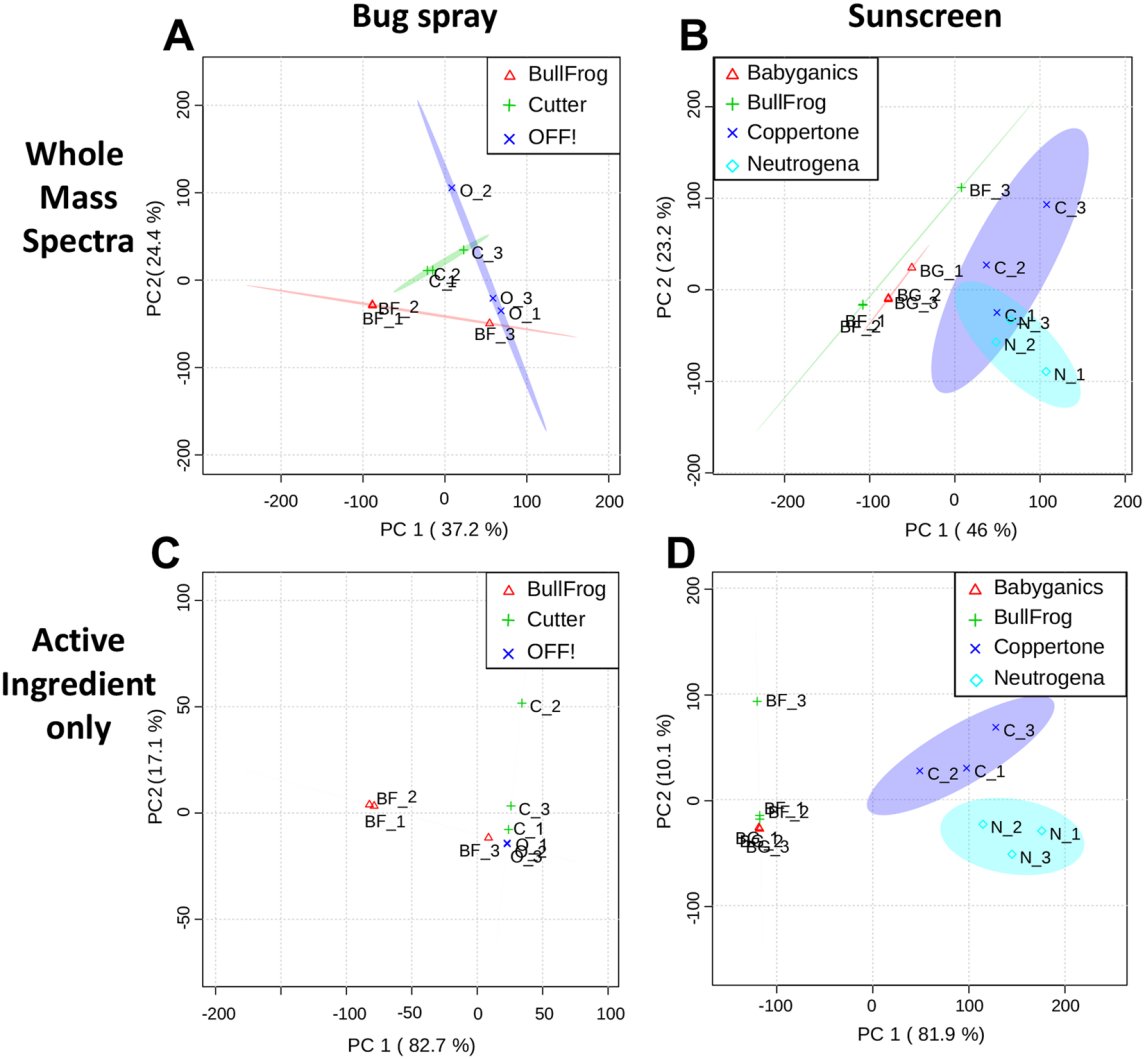
PCA scatter plots of (**A,C**) bug spray and (**B,D**) sunscreen containing fingerprints using either entire mass spectrum (**A,B**) or only active ingredients (**C,D**).

To further investigate the ability to distinguish brands, we applied principal component analysis (PCA) to the bug spray and sunscreen data. Initially we applied PCA to the entire spectrum of the bug spray and sunscreen replicate fingerprint data. Normalized intensities in the *m/z* range of 150 to 500 with more than one percent ion signals were submitted to MetaboAnalyst^26^. As shown in Fig. 3A,B, there is some separation between the brands in both bug spray and sunscreen, but the separation is not complete. Specifically, in Fig. 3B overlap is seen in sunscreen between Coppertone and Neutrogena. This was expected as both Coppertone and Neutrogena contain the same five active ingredients with some differences in abundance.

To avoid the effect of sample variation due to non-active ingredients, another PCA analysis was performed with active ingredients only, as shown in Fig. 3C,D. As expected, the separation is much better than the full mass spectra suggesting the importance of a targeted approach. When only active ingredients are compared for their normalized signal intensities as shown in Fig. S1 for bug spray and Fig. 2B for sunscreen, the separation is very clear between the brands. This emphasizes the importance of establishing a mass list of known compounds when studying exogenous compounds in latent fingerprints for lifestyle markers. In some sense, it is similar to typical metabolomics workflow. Untargeted statistical analysis is commonly performed, such as PCA or hierarchical clustering analysis, when compounds of interest are not well established^27^. Quantitative profiling of selected target compounds, however, gives better insight about the biological processes once characteristic compounds are identified. For the rest of this paper, our effort will be focused on finding characteristic marker compounds.

### Food Oils

**Figure 4:**
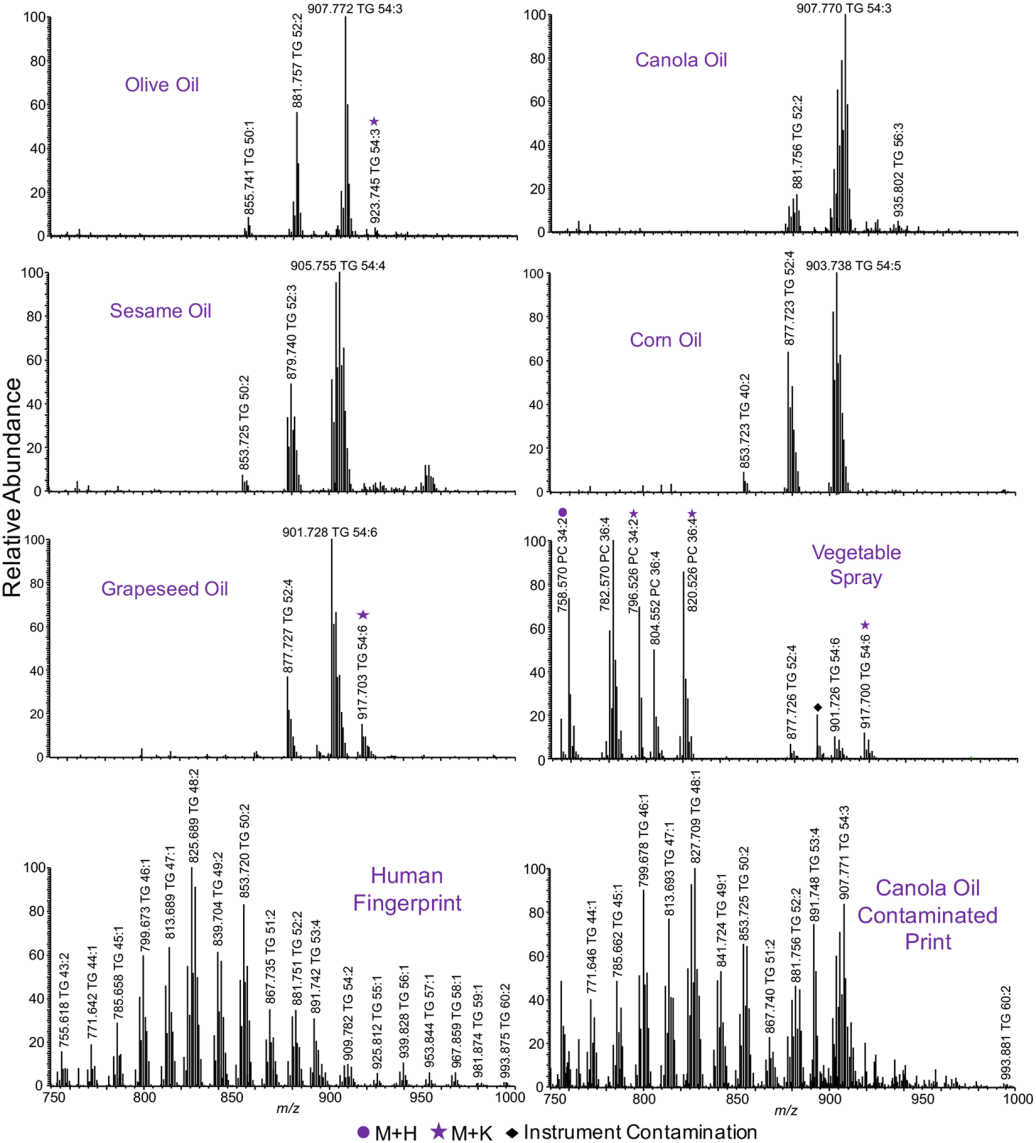
Representative positive mass spectra of five plant-based food oils, a vegetable oil spray, and human fingerprints with and without contamination by MALDI-MSI with DHB as the matrix. All non-labeled TGs are observed as sodium adducts and others are as proton or potassium adducts as labeled.

Another set of compounds likely to be present in a latent fingerprint are food oils, from cooking or eating. TGs are the main constituent of cooking oils, mostly produced from plant seeds. Human fingerprints also contain TGs, secreted from sweat glands, but those from plants have distinct patterns of fatty acyl chains. Fig. 4 compares TG profiles of five cooking oils (olive, canola, sesame, corn, and grapeseed), a vegetable oil spray, and a human fingerprint. As expected, each oil shows patterns distinguishable from human TGs and other cooking oils. The most abundant TG species in olive and canola oil is TG 54:3 as a sodiated adduct at *m/z* 907. TG 54:4 at *m/z* 905, TG 54:5 at *m/z* 903, and TG 54:6 at *m/z* 901 are the most abundant species in sesame, corn, and grapeseed oil, respectively, all as sodium adducts. The four abundant TGs in food oil are present only in minimal abundance in human fingerprints. Olive oil has very narrow unsaturation patterns, dominated by TG 54:3 and TG 52:2. In contrast, other oils have broad unsaturation patterns, mostly TG 52:1-4 and TG 54:2-6, with only subtle differences, but clearly distinguishable by the most abundant TG species. In canola oil the abundances of TG 54:6/54:5/54:4/54:3 are 30/70/80/100 compared to 50/95/100/65, 85/100/65/25, and 100/65/40/15 in sesame, corn, and grapeseed oil respectively. These TG profiles are similar to those reported by others^28–30^.

Vegetable oil spray contained soybean lecithin in addition to soybean oil, which led to the presence of phosphatidylcholines (PCs) along with the TGs. Vegetable oil spray is easily identified in fingerprints due to multiple unique TGs and PCs. Other oils, such as coconut oil, can also be easily distinguished as shown in Fig. S2. The mass spectrum of coconut oil is dominated by DGs and TGs in the *m/z* 500–800 region^29^. TGs of human origin appear in the broad mass range of *m/z* 750–1000, TG 43:x to 60:x, as shown in Fig. 4, and are quite different from plant oils. TG 52:x and 54:x series overlap with plant TGs, but they are much less abundant, whereas the most abundant TG 48:x series in fingerprints is absent in plant oils. As also shown in Fig. 4, TGs in human and plant oil can be easily distinguished in a food oil contaminated fingerprint, and the identity of the oil species could be easily determined as canola oil based on the most abundant TG and the broader unsaturation pattern. Previous work by Ng *et al*. showed that vegetable oils from different manufacturers displayed similar TG profiles, despite differences in oil processing, demonstrating reproducible TG profiles for source determination^30^. Food oil can tell more of an individual’s story, such as which cooking oil was used when they cooked or ate prior to leaving a fingerprint behind.

### Alcohol Compounds

**Figure 5:**
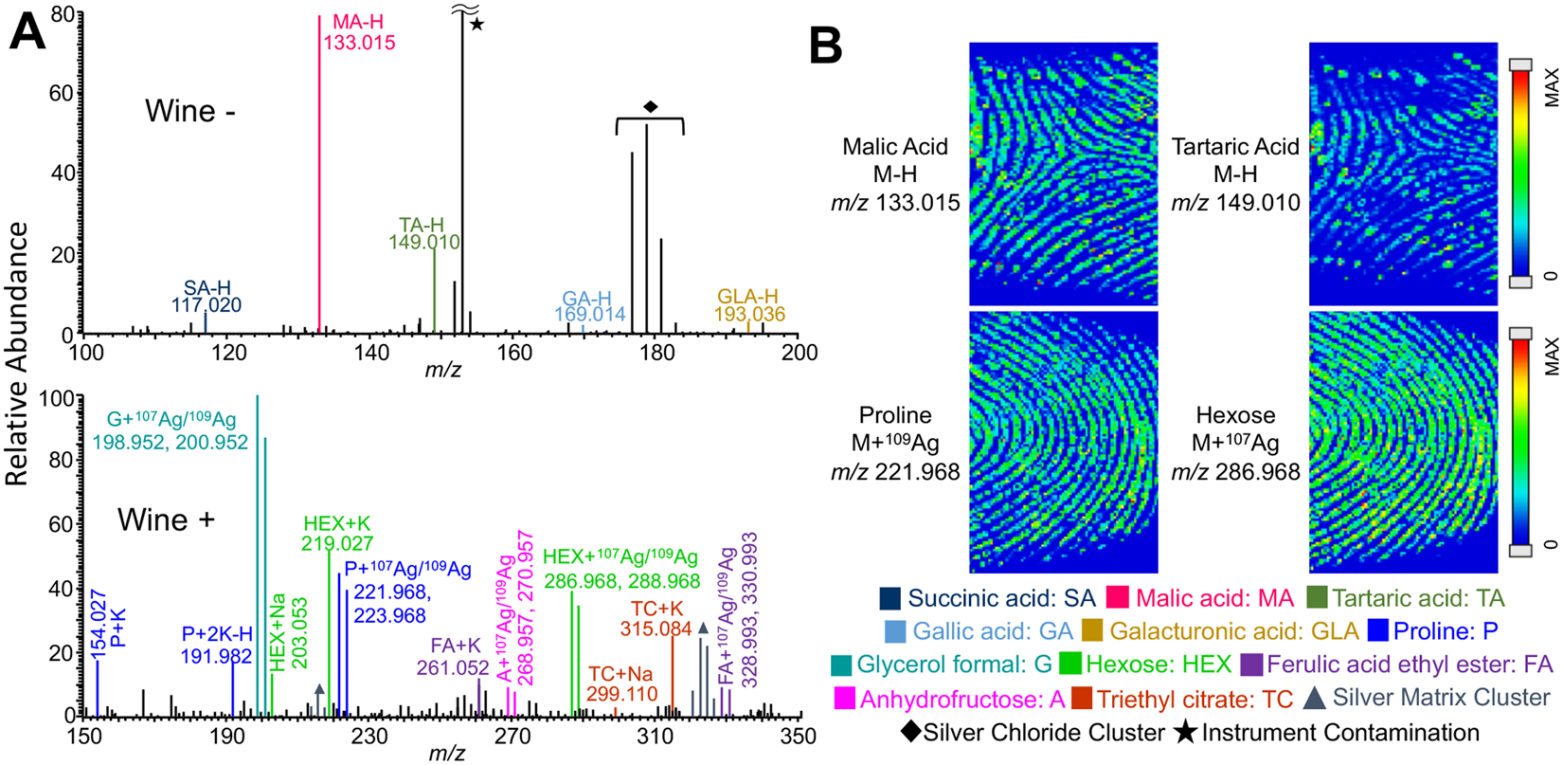
(**A**) Representative negative and positive mode mass spectra of wine by MALDI-MSI with silver sputter. (**B**) Chemical images of wine related compounds from contaminated fingerprints.

Whether it be from a consumption situation or the presence in a bar, identifying compounds related to alcoholic beverages in a fingerprint can provide a piece of a person’s lifestyle. The analysis of wine proved to be the most informative in negative mode, due to several organic acids known to be present in wine, including gallic, tartaric, succinic, malic, and galacturonic acids (Fig. 5A). Tartaric acid and malic acid are the most abundant acids in wine^31^, and the presence of these acids, especially tartaric acid, is a strong indication of wine. In positive mode, proline, glycerol formal, hexose, and anhydrofructose were the most abundant wine related peaks, but these compounds are not exclusive to wine. Although these compounds are not individually exclusive, the combination of these compounds may suggest the potential presence of wine. Fingerprint images of malic acid, tartaric acid, proline, and hexose are included in Fig. 5B. If wine related compounds are confirmed in a latent print, they would indicate a wine drinker as the fingerprint source or suggest the person was recently in an establishment that serves wine.

Other alcohol beverages, such as beer and whiskey, were also investigated for their potential as exogenous compounds in latent fingerprints. Both beer and whiskey were dominated by sugars, lacking unique compounds for identification (Fig. S3A). When PCA analysis is performed between beer and whiskey (Fig. S3B) or among three alcohols including wine (Fig. S3C), separation could be made between alcohol beverages, suggesting they could be distinguished in a similar way to sunscreen (Fig. 2B). However, major compounds identified in beer or whiskey could have come from any number of sources, making it impossible to relate back to beer and whiskey specifically. For example, triethyl citrate, glucose (hexose), and sucrose (hexose_2_) are also found in citrate fruits (see next section) as well as bug spray and sunscreen fingerprints (Figs. 1A and 2A).

### Citrus Fruits

**Figure 6:**
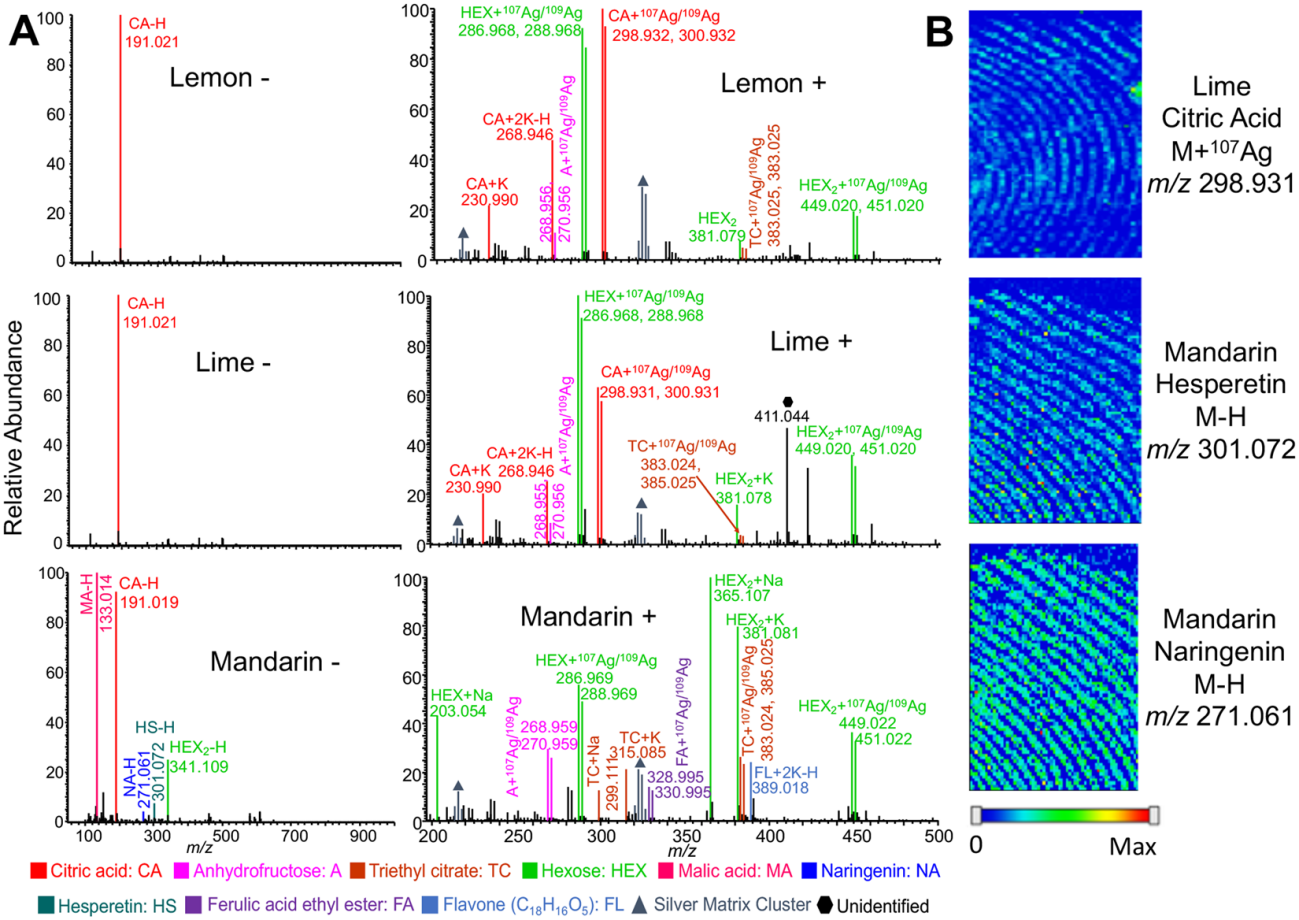
(**A**) Representative mass spectra of lemon, lime, and mandarin in negative and positive modes by MALDI-MSI with silver sputter. (**B**) Three chemical images related to citrus fruits.

Citrus fruits are commonly eaten using bare hands, likely leading to compound transfer onto the fingers. The chemical compounds in mandarins, lemons, and limes were explored. While multiple compounds were identified in the mass spectra (Fig. 6A), the presence of citric acid in combination with abundant sugars are a good indication of a citrus fruit. Citric acid is present in all three fruits, but the abundance is consistently higher (>5 times) in the lemon and the lime, which instead have lower abundance of sugars than in the mandarin. The mandarin could be differentiated based on the higher intensity of sugars, and the presence of naringenin, hesperetin, and malic acid in negative mode. In Fig. 6B chemical images of three citrus related compounds from two different fingerprints are displayed. The type of citrus fruit leading to compounds in a latent print could be used for comparison with those in an individual’s habitual space.

### Mock Experiment and Multiplex Fingerprint Imaging

To further explore the usefulness of lifestyle markers, our previously developed multiplex MSI method was applied to the analysis of fingerprints. This approach can simultaneously acquire fingerprint MS images in HRMS as well as thousands of MS/MS spectra of potential marker compounds for confident identification. It can be accomplished in a single data acquisition by dividing each imaging pixel into multiple pixels with different MS events. A simultaneous analysis is crucial when only a single print is recovered from a crime scene. Often, useful and unique exogenous compounds are of low signal intensity. We made a mass list of unique lifestyle marker compounds for bug sprays, sunscreens, food oils, alcohols, and citrus fruits. This precursor mass list was included into the data acquisition software to initiate their automatic fragmentation when the precursor mass was detected. Abundant contamination peaks, matrix peaks, or other peaks of non-interest are added in the exclusion list in the multiplex MS method. The combination of a precursor mass list and exclusion list lead to improved detection, fragmentation, and identification of low abundance useful exogenous compounds. MS/MS for low abundance compounds may not otherwise be acquired.

Table S2 summarizes a list of the key compounds studied in this paper, excluding TGs and hexose, and their major product ions in MS/MS. The list also includes low intensity compounds that were not consistently identified in every sample but were confirmed through MS/MS in multiplex MSI. When multiple adducts are detected for a compound (e.g., [M + H]^+^, [M + Na]^+^, [M + K]^+^, [M + ^107^Ag]^+^, [M + ^109^Ag]^+^), an adduct form with the most efficient MS/MS was included in the table. Compounds in a latent fingerprint can be compared to the table and identification can be made based on accurate mass and MS/MS.

**Figure 7:**
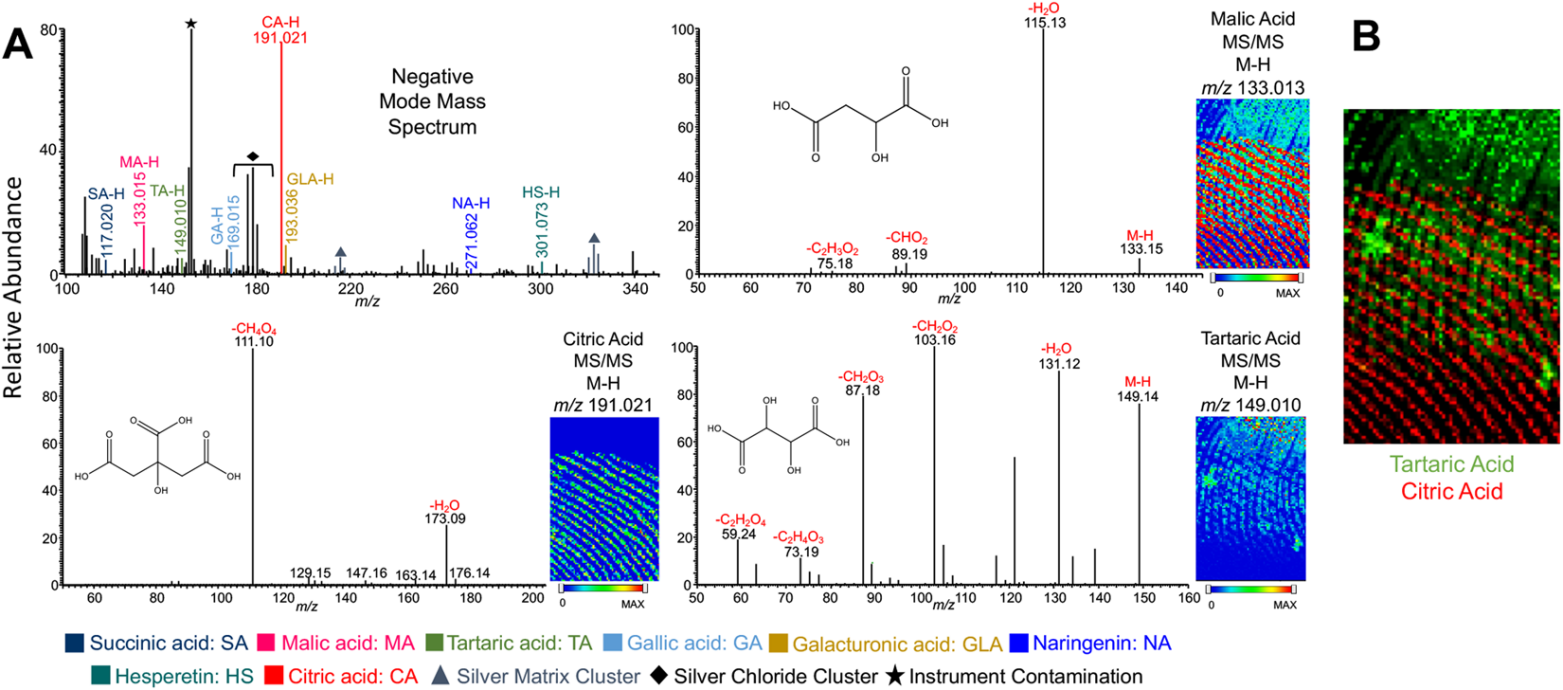
(**A**) Negative mode mass spectrum and MS/MS spectra of three exogenous compounds from a mock experiment, obtained in a single multiplex acquisition by MALDI-MSI with silver sputter. (**B**) Chemical images of two compounds differentiating the overlapped fingerprints.

As a mock experiment to demonstrate how multiplex MS imaging can be used to identify lifestyle markers from latent fingerprints, two overlapping fingerprints were prepared with different sources of exogeneous compounds. One print was purposefully contaminated with wine while the other was contaminated from a mandarin fruit. In Fig. 7A a negative mode mass spectrum and the related mass spectral images of the two overlapped fingerprints are displayed. Two compounds, citric acid and tartaric acid, were present in different prints and the corresponding exact masses were used to individually extract the two prints. Citric acid originated from the mandarin fruit, while the tartaric acid was from the wine contaminated fingerprint. Malic acid had previously been identified in both mandarin fruit and wine, and appeared in both but with a higher abundance in the mandarin contaminated print. Confident identification cannot be made with accurate mass only. The multiplex method allowed MS/MS of citric acid, tartaric acid, and malic acid from the precursor mass list, which matches with the MS/MS library (Table S2). Citric acid is a good indication of citrus fruit but not a definitive clue for mandarin; however, the existence of naringenin and hesperetin in the MS spectrum gives confidence it is from mandarin. A combined chemical image of two compounds, citric acid and tartaric acid, was extracted and displays the distinct prints each compound is related to (Fig. 7B). The fingerprint images and multiple MS/MS spectra were obtained in a single data acquisition, demonstrating the efficiency and usefulness of the multiplex MSI technique. The combination of ridge detail and confirmed fragmentation pattern are strong evidence for detailed chemical information about the individual depositing the fingerprint.

## Conclusions

In addition to the ridge pattern, the exogenous chemical compounds present in latent fingerprints provide hidden evidentiary value. Products applied, food and beverages consumed, and various environmental contacts will lead to a variety of chemical compounds in a latent fingerprint that can provide lifestyle information of an individual. MALDI-MSI proved to be efficient at ionizing a broad range of exogenous compounds in conjunction with silver sputter coating as a matrix. The simultaneous collection of a HRMS image along with MS/MS through our multiplex capabilities allowed spatial determination and confident compound identification in a single fingerprint analysis. It is important to note that the work presented here demonstrates proof-of-concept for exogenous compound source determination in latent prints that can be used to provide lifestyle information of the individuals. More work needs to be done before applying to real forensic cases, such as a large population study accompanied with statistical analysis or aged (non-ideal) sample studies. While the exogenous compounds studied here are not exhaustive, the further development of an exogenous compound database combined with multiplex MSI will facilitate lifestyle determination based on fingerprint chemical compounds.

## Materials and Methods

### Internal Review Board Approval

The methods and analyses carried out in this work were approved by and in accordance with the Iowa State University Internal Review Board guidelines, specifically the collection of fingerprints for analysis. Experiments were conducted using the researchers own fingerprints, so informed consent documents were not required.

### Sample Preparation and Matrix Application

All consumer products were available over the counter and purchased from a local retailer. The bug spray and sunscreen were applied per product instructions, and a fingerprint was then deposited on a glass slide pre-cleaned with methanol. Citrus fruits were handled as if being consumed or cut into slices prior to handling and fingerprint deposition. All alcohol and food oil samples were touched to mimic a spill or the handling of food-ware before making a fingerprint on the glass slide. Standards for ethyl palmitate, ethyl myristate, glycerol, hesperetin, hesperidin, and limonene were purchased from Sigma (St. Louis, MO, USA) for the best purity available and prepared at a concentration of 100 μM in methanol, and 5 µL were spotted onto a glass slide. A triethyl citrate standard was purchased from TCI America (Portland, OR, USA) and prepared using the above-mentioned procedure. All prints and standards were allowed to dry under ambient conditions before matrix was applied.

Organic matrices, DHB and CHCA (Sigma), were sublimated following previously published guidelines^32^. Gold and silver targets (Ted Pella; Redding, CA, USA) were sputter coated (108 Auto Sputter Coater, Ted Pella) at 40 mA for 10 and 5 seconds, respectively.

### Instrumentation, Data Acquisition, and Data Analysis

A linear ion trap-Orbitrap mass spectrometer with a MALDI ion source (MALDI LTQ-Orbitrap Discovery, Thermo Scientific; San Jose, CA, USA) was coupled with a 355 nm Nd:YAG laser (UVFQ, Elforlight Ltd.; Daventry, UK). Imaging and profiling of fingerprint samples was done with a 100 μm raster step, 10 laser shots per raster step, and a 30 μm laser spot size. Scans were collected for *m/z* 50–1000 (mass resolution of 30,000 at *m/z* 400). For multiplex MSI, a spiral raster step was employed as previously described^16,17^. A precursor mass list and an exclusion mass list were imported into the Excalibur data acquisition program (Thermo Scientific) for the efficient precursor ion selection in data-dependent MS/MS scans. Collision energies were broadly assigned depending on the precursor mass range for optimal fragmentation (75 for masses greater than 300 Da, and 125 for masses less than 300 Da). An isolation width of 2.0 Da was used. Positive and negative ion mode were employed for profiling, imaging, and multiplex MSI.

QualBrowser (Thermo Scientific) software was used to average and export all mass spectra. Chemical fingerprint images were generated using ImageQuest (Thermo Scientific) software with a mass tolerance of ±0.05 Da. The intensity scale of each image was arbitrarily adjusted based on the individual compound intensity to produce quality images. Each compound was confirmed using a standard and/or database comparison for fragmentation patterns. To compare the intensity of active ingredients among distinct brands, the mass spectra were exported to an Excel spreadsheet using QualBrowser. The intensities of all the adducts were summed for each ingredient and normalized to the most abundant compound in each data set. The three replicates were averaged, and the standard deviation was calculated. The averages and standard deviations were plotted for comparison and included in Fig. S1 for bug spray and Fig. 2B for sunscreen.

### Principal Component Analysis

Data for PCA was extracted using the QualBrowser software. The data was averaged over the *m/z* range 150–500, as most relevant compounds are found in this region. Each peak was normalized to the most abundant peak in the mass region. Peaks with a normalized intensity of less than one percent were not extracted. Three replicates of each brand being studied were included in the PCA. The mass list was then uploaded to MetaboAnalyst for statistical analysis^26^. Scores plots for both bug spray and sunscreen mass spectra were included in Fig. 3. For a targeted approach, peak intensity tables of the active ingredients were uploaded rather than the entire mass list.

### Data availability statement

The datasets generated and analyzed during the current study are available from the corresponding author upon reasonable request.

## Electronic supplementary material

Supplementary Information

## References

[1] Cadd SJ (2015). Extraction of fatty compounds from fingerprints for GCMS analysis. Anal. Methods.

[2] Croxton RS, Baron MG, Butler D, Kent T, Sears VG (2006). Development of a GC-MS method for the simultaneous analysis of latent fingerprint components. J. Forensic Sci..

[3] De Puit M, Ismail M, Xu X (2014). LCMS Analysis of Fingerprints, the Amino Acid Profile of 20 Donors. J. Forensic Sci..

[4] Muramoto S, Sisco E (2015). Strategies for Potential Age Dating of Fingerprints Through the Diffusion of Sebum Molecules on a Nonporous Surface Analyzed Using Time-of-Flight Secondary Ion Mass Spectrometry. Anal. Chem..

[5] Bailey MJ (2013). Enhanced imaging of developed fingerprints using mass spectrometry imaging. Analyst.

[6] Sisco E, Demoranville LT, Gillen G (2013). Evaluation of C60 secondary ion mass spectrometry for the chemical analysis and imaging of fingerprints. Forensic Sci. Int..

[7] Bailey MJ (2015). Rapid detection of cocaine, benzoylecgonine and methylecgonine in fingerprints using surface mass spectrometry. Analyst.

[8] Zhou, Z. & Zare, R. N. Personal Information from Latent Fingerprints Using Desorption Electrospray Ionization Mass Spectrometry andMachine Learning. *Anal. Chem*. acs.analchem. 6b04498, 10.1021/acs.analchem.6b04498 (2017). 10.1021/acs.analchem.6b04498 28194988

[9] Guinan T, Della Vedova C, Kobus H, Voelcker NH (2015). Mass spectrometry imaging of fingerprint sweat on nanostructured silicon. Chem. Commun..

[10] Forbes TP, Sisco E (2014). Chemical imaging of artificial fingerprints by desorption electro-flow focusing ionization mass spectrometry. Analyst.

[11] Kaplan-Sandquist K, LeBeau MA, Miller ML (2014). Chemical analysis of pharmaceuticals and explosives in fingermarks using matrix-assisted laser desorption ionization/time-of-flight mass spectrometry. Forensic Sci. Int..

[12] O’Neill, K. C. & Lee, Y. J. Effect of Aging and Surface Interactions on the Diffusion of Endogenous Compounds in Latent Fingerprints Studied by Mass Spectrometry Imaging. *J. Forensic Sci*. 1–6, 10.1111/1556-4029.13591 (2017). 10.1111/1556-4029.13591 28691753

[13] Beinsen, A. & Abel, B. Matrix Assisted and Matrix Free Mass Spectrometric Imaging of Latent Fingermarks. *Curr. Top. Anal. Chem*. (2011).

[14] Bailey MJ (2012). Chemical Characterization of Latent Fingerprints by Matrix-Assisted Laser Desorption Ionization, Time-of-Flight Secondary Ion Mass Spectrometry, Mega Electron Volt Secondary Mass Spectrometry, Gas Chromatography/Mass Spectrometry, X-ray Photoelectron Spec. Anal. Chem..

[15] Francese, S. In *Advances in MALDI and Laser-Induced Soft IonizationMass Spectrometry*(ed. Cramer, R.) 93–128, 10.1007/978-3-319-04819-2 (Springer International Publishing, 2016).

[16] Perdian DC, Lee YJ (2010). Imaging MS Methodology for More Chemical Information in Less Data Acquisition Time Utilizing a Hybrid Linear Ion Trap- Orbitrap Mass Spectrometer. Anal. Chem..

[17] Yagnik GB, Korte AR, Lee YJ (2013). Multiplex mass spectrometry imaging for latent fingerprints. J. Mass Spectrom..

[18] Ferguson LS (2012). Direct detection of peptides and small proteins in fingermarks and determination of sex by MALDI mass spectrometry profiling. Analyst.

[19] Wolstenholme R, Bradshaw R, Clench MR, Francese S (2009). Study of latent fingermarks by matrix-assisted laser desorption/ionisation mass spectrometry imaging of endogenous lipids. Rapid Commun. Mass Spectrom..

[20] Emerson B, Gidden J, Lay JO, Durham B (2011). Laser desorption/ionization time-of-flight mass spectrometry of triacylglycerols and other components in fingermark samples. J. Forensic Sci..

[21] Lauzon N, Dufresne M, Chauhan V, Chaurand P (2015). Development of laser desorption imaging mass spectrometry methods to investigate the molecular composition of latent fingermarks. J. Am. Soc. Mass Spectrom..

[22] Groeneveld G, de Puit M, Bleay S, Bradshaw R, Francese S (2015). Detection and mapping of illicit drugs and their metabolites in fingermarks by MALDI MS and compatibility with forensic techniques. Sci. Rep..

[23] Bouslimani A (2016). Lifestyle chemistries from phones for individual profiling. Proc. Natl. Acad. Sci. USA.

[24] Petras, D. *et al*. Mass Spectrometry-Based Visualization of Molecules Associated with Human Habitats. *Anal. Chem*. acs.analchem. 6b03456, 10.1021/acs.analchem.6b03456 (2016). 10.1021/acs.analchem.6b03456 PMC6326777 27732780

[25] Asbury GR, Al-Saad K, Siems WF, Hannan RM, Hill HH (1999). Analysis of triacylglycerols and whole oils by matrix-assisted laser desorption/ionization time of flight mass spectrometry. J. Am. Soc. Mass Spectrom..

[26] Xia J, Wishart DS (2016). Using metaboanalyst 3.0 for comprehensive metabolomics data analysis. Curr. Protoc. Bioinforma..

[27] Dettmer K, Aronov PA, Hammock BD (2007). Mass spectrometry-based metabolomics. Mass Spectrom. Rev..

[28] Calvano CD, De Ceglie C, D’Accolti L, Zambonin CG (2012). MALDI-TOF mass spectrometry detection of extra-virgin olive oil adulteration with hazelnut oil by analysis of phospholipids using an ionic liquid as matrix and extraction solvent. Food Chem..

[29] Schiller J, Sub R, Petkovic M, Arnold K (2002). Triacylclycerol analysis of vegetable oils by matrix-assisted laser desorption and ionization time-of-flight (MALDI-TOF) mass spectrometry and 31P NMR Spectroscopy. J. Food Lipids.

[30] Ng TT, So PK, Zheng B, Yao ZP (2015). Rapid screening of mixed edible oils and gutter oils by matrix-assisted laser desorption/ionization mass spectrometry. Anal. Chim. Acta.

[31] Rajković M, Novaković ID, Petrović A (2007). Determination of titratable acidity in white wine. J. Agric. Sci..

[32] Hankin JA, Barkley RM, Murphy RC (2007). Sublimation as a Method of Matrix Application for Mass Spectrometric Imaging. J. Am. Soc. Mass Spectrom..

