# Supplementary material for: Revealing Individual Lifestyles through Mass Spectrometry Imaging of Chemical Compounds in Fingerprints
Source: Sci Rep. 2018 Mar 26;8:5149. doi: 10.1038/s41598-018-23544-7 (PMC5979955; doi:10.1038/s41598-018-23544-7)
Supplement: Supplementary file 1 — Supplementary Information [file 41598_2018_23544_MOESM1_ESM.pdf]

Supplemental Information for:

Revealing Individual Lifestyles through Mass Spectral Imaging of Chemical Compounds in Fingerprints

Paige Hinners[1], Kelly C. O'Neill[1], and Young Jin Lee[1*]

[1] Department of Chemistry, Iowa State University, Ames, IA, 50011, USA

*Corresponding author: Dr. Young Jin Lee

0035A Roy J Carver Co-Lab

1111 WOI Road

Ames, IA 50011-3650

Tel: 515-294-1235

Email: yjlee@iastate.edu

Table S1. The active ingredient list of each sunscreen brand. Y=Yes, N=No.

Compound/Brand	Neutrogena	Coppertone	BullFrog	Babyganics
Avobenzone	Y	Y	N	N
Homosalate	Y	Y	N	N
Octisalate	Y	Y	Y	Y
Octocrylene	Y	Y	Y	N
Oxybenzone	Y	Y	Y	N
Octinoxate	N	N	Y	Y

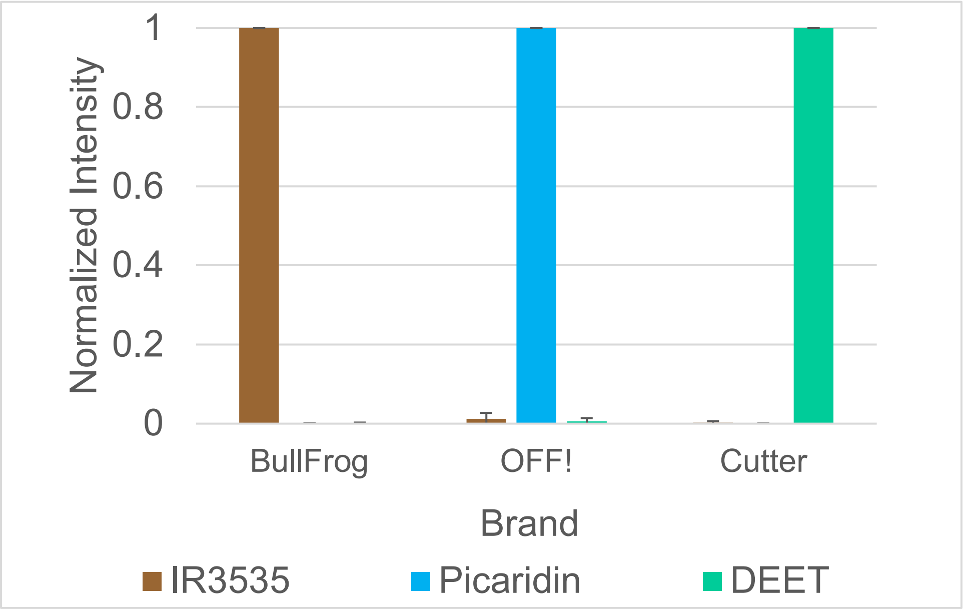

Fig. S1. Comparison of each active ingredient across the three bug spray brands. The adduct intensities are summed for each active ingredient and normalized to the most abundant compound. Error bars show the standard deviation from three replicates.

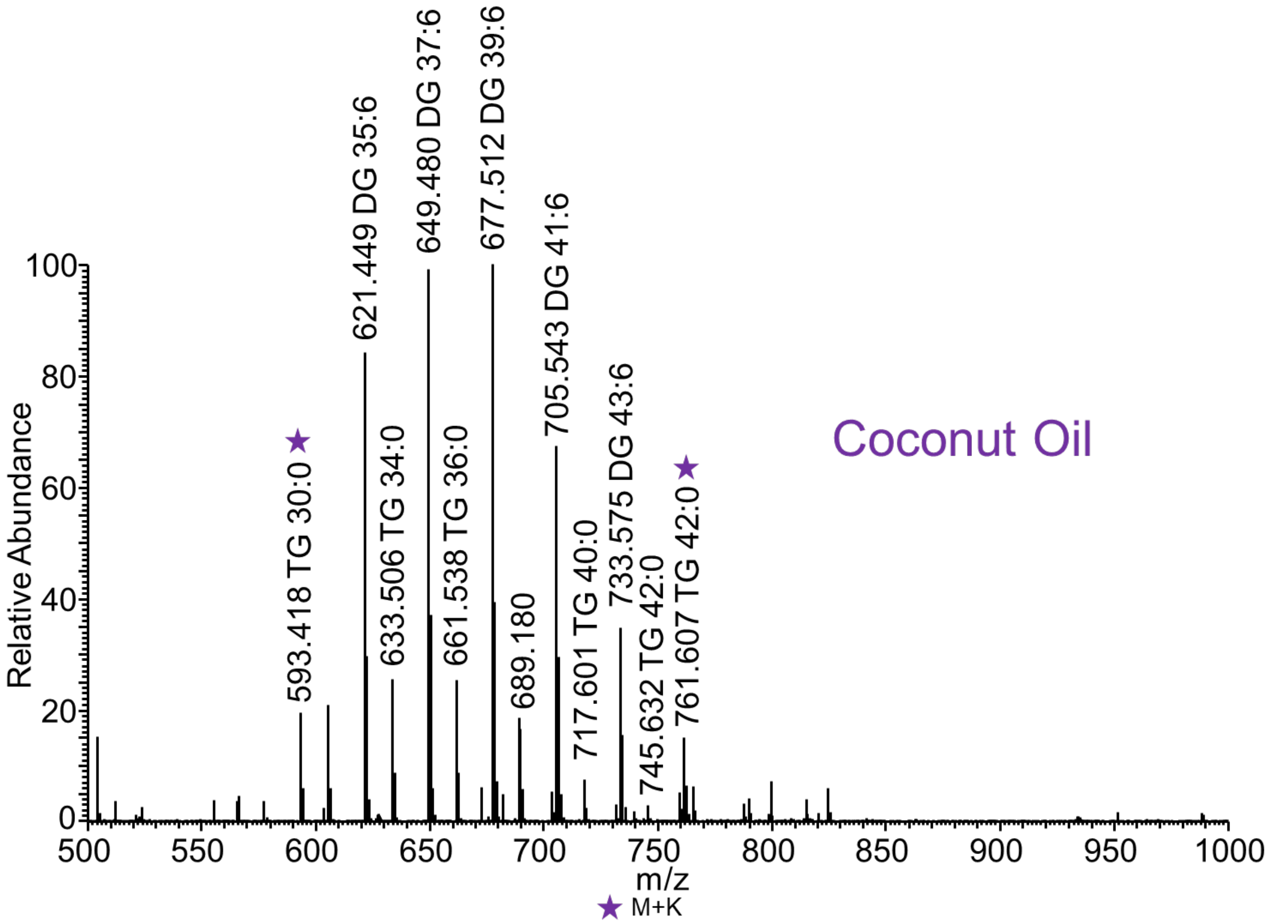

Fig. S2. The TGs and DGs indicative of coconut oil are in the *m/z* range of 500-800, a lower mass range than most plant-based food oils. Note that all DGs present contain five oxygens, therefore they are considered a natural DG rather than MALDI generated.

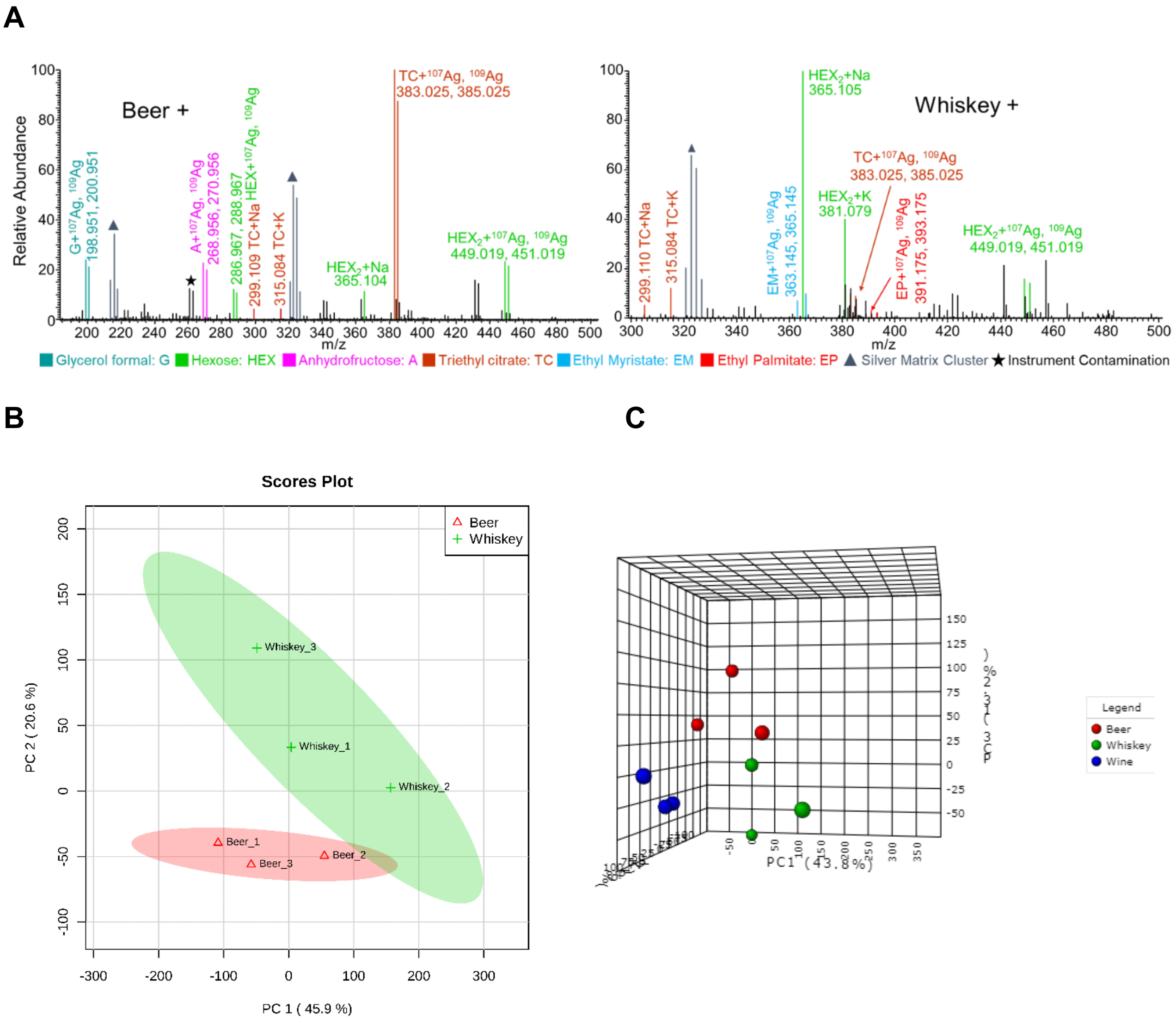

Fig. S3. (A) The positive mode mass spectra of beer and whiskey using silver matrix for MALDI-MSI. (B) PCA analysis of beer and whiskey only. (C) PCA analysis of beer, whiskey, and wine in 3D display.

Table S2. A list of exact *m/z* values for exogenous compounds, as well as the corresponding fragment ions for confident compound identification.

Compound	Source	Mode	MALDI-MS	MALDI-MS/MS
			Adduct	Product Ions
Avobenzone $C_{20}H_{22}O_3$	Sunscreen	+	311.164 $[M+H]^+$	293, 161, 135
Homosalate $C_{16}H_{22}O_3$	Sunscreen	+	369.061 $[M+^{107}Ag]^+$	354, 341, 249
Octinoxate $C_{18}H_{26}O_3$	Sunscreen	+	291.195 $[M+H]^+$	276, 179, 121
Octisalate $C_{15}H_{22}O_3$	Sunscreen	+	273.146 $[M+Na]^+$	255, 137
Octocrylene $C_{24}H_{27}NO_2$	Sunscreen	+	384.193 $[M+Na]^+$	324, 272, 228
Oxybenzone $C_{14}H_{12}O_3$	Sunscreen	+	229.086 $[M+H]^+$	51, 105
DEET $C_{12}H_{17}NO$	Bug spray	+	192.138 $[M+H]^+$	174, 119
IR3535 $C_{11}H_{21}NO_3$	Bug spray	+	216.159 $[M+H]^+$	170
Picaridin $C_{12}H_{23}NO_3$	Bug spray	+	230.175 $[M+H]^+$	212, 174
Ethyl myristate $C_{16}H_{32}O_2$	Wine	+	363.145 $[M+^{107}Ag]^+$	345, 335, 317
Ethyl palmitate $C_{18}H_{36}O_2$	Wine	+	391.176 $[M+^{107}Ag]^+$	373, 345, 331
Galacturonic acid $C_6H_{10}O_7$	Wine	-	193.034 $[M-H]^-$	131, 113, 89
Gallic acid $C_7H_6O_5$	Wine	-	169.013 $[M-H]^-$	151, 141, 125
Glycerol formal $C_4H_8O_3$	Wine	+	200.951 $[M+^{109}Ag]^+$	183, 109
Proline $C_5H_9NO_2$	Wine	+	221.968 $[M+^{107}Ag]^+$	176, 114
Succinic acid $C_4H_6O_4$	Wine	-	117.018 $[M-H]^-$	99, 73
Tartaric acid $C_4H_6O_6$	Wine	-	149.008 $[M-H]^-$	131, 103, 87, 73, 59
Anhydrofructose $C_6H_{10}O_5$	Wine, citrus	+	268.956 $[M+^{107}Ag]^+$	251, 191
Ferulic acid ethyl ester $C_{12}H_{14}O_4$	Wine, citrus	+	328.994 $[M+^{107}Ag]^+$	311, 269, 255
Malic acid $C_4H_6O_5$	Wine, citrus	-	133.013 $[M-H]^-$	115, 89, 75
Triethyl citrate $C_{12}H_{14}O_4$	Multiple	+	383.026 $[M+^{107}Ag]^+$	355, 295, 263
Citric acid $C_6H_8O_7$	Citrus	-	191.019 $[M-H]^-$	173, 111
Hesperidin $C_{28}H_{34}O_{15}$	Citrus	+	717.094 $[M+^{107}Ag]^+$	571, 409
Hesperetin $C_{16}H_{14}O_6$	Citrus	+	408.984 $[M+^{107}Ag]^+$	301, 259
Limonene $C_{10}H_{16}$	Citrus	+	243.030 $[M+^{107}Ag]^+$	175, 135, 107
Naringenin $C_{15}H_{12}O_5$	Citrus	-	271.061 $[M-H]^-$	177, 151, 119
Nobiletin $C_{21}H_{22}O_8$	Citrus	+	403.139 $[M+H]^+$	388, 373, 342
Tangeretin $C_{20}H_{20}O_7$	Citrus	+	373.128 $[M+H]^+$	358, 343, 312
